# Lipopeptides from *Bacillus velezensis* ZLP-101 and their mode of action against bean aphids *Acyrthosiphon pisum Harris*

**DOI:** 10.1186/s12866-024-03378-2

**Published:** 2024-06-29

**Authors:** Qiuyue Liu, Wenya Zhao, Wenya Li, Feiyan Zhang, Yana Wang, Jiangping Wang, Yumeng Gao, Hongwei Liu, Liping Zhang

**Affiliations:** 1 Institute of Biology, Hebei Academy of Science, Shijiazhuang, 050081 PR China; 2 Hebei Normal University, Shijiazhuang, 050024 PR China; 3 Main Crops Disease of Microbial Control Engineering Technology Research Center in Hebei Province, Shijiazhuang, 050081 PR China

**Keywords:** *Bacillus velezensis* ZLP-101, Genome sequence, *Acyrthosiphon pisum Harris*, Insecticidal mechanism, Lipopeptides

## Abstract

**Background:**

Natural products are important sources for the discovery of new biopesticides to control the worldwide destructive pests *Acyrthosiphon pisum Harris*. Here, insecticidal substances were discovered and characterized from the secondary metabolites of the bio-control microorganism *Bacillus velezensis* strain ZLP-101, as informed by whole-genome sequencing and analysis.

**Results:**

The genome was annotated, revealing the presence of four potentially novel gene clusters and eight known secondary metabolite synthetic gene clusters. Crude extracts, prepared through ammonium sulfate precipitation, were used to evaluate the effects of strain ZLP-101 on *Acyrthosiphon pisum Harris* aphid pests via exposure experiments. The half lethal concentration (LC50) of the crude extract from strain ZLP-101 against aphids was 411.535 mg/L. Preliminary exploration of the insecticidal mechanism revealed that the crude extract affected aphids to a greater extent through gastric poisoning than through contact. Further, the extracts affected enzymatic activities, causing holes to form in internal organs along with deformation, such that normal physiological activities could not be maintained, eventually leading to death. Isolation and purification of extracellular secondary metabolites were conducted in combination with mass spectrometry analysis to further identify the insecticidal components of the crude extracts. A total of 15 insecticidal active compounds were identified including iturins, fengycins, surfactins, and spergualins. Further insecticidal experimentation revealed that surfactin, iturin, and fengycin all exhibited certain aphidicidal activities, and the three exerted synergistic lethal effects.

**Conclusions:**

This study improved the available genomic resources for *B. velezensis* and serves as a foundation for comprehensive studies of the insecticidal mechanism by *Bacillus velezensis* ZLP-101 in addition to the active components within biological control strains.

**Supplementary Information:**

The online version contains supplementary material available at 10.1186/s12866-024-03378-2.

## Introduction

The heavy use of chemical pesticides to achieve crop protection has led to increased concerns about their possible adverse effects on biodiversity, environmental pollution, and human health [1]. Biopesticides, which are living organisms or products derived from living organisms, are considered promising alternatives to traditional pesticides [2]. Bacillus is an important biological control microorganism that can prevent plant disease and promote plant growth [3]. In particular, *Bacillus velezensis*, which is a non-pathogenic bacterium that is naturally ubiquitously distributed and produces bioactive compounds, also promotes plant growth [4]. Consequently, *B. velezensis* has been used as an agricultural biological control agent [5, 6]. The development of high-throughput and low-cost sequencing technologies have led to the widespread use of genomic-based analyses to comprehensively understand the metabolites and physiologies of microorganisms [5, 7, 8]. For example, analyses of the increasingly available genomes for different strains of *B. velezensis* have demonstrated significant differences in gene clusters involved in secondary metabolite production among strains, to the extent that the active substances produced by some strains may be completely different [3, 9]. Further the whole-genome DNA·of *B. velezensis* ZLP-101 was sequenced to guide compound isolation. Thus, a more thorough understanding of the *B. velezensis* ZLP-101 genome can improve our understanding of the ability of *B. velezensis* to produce various secondary metabolites and provide a framework for further analysis of biological control.

Bacillus can produce various secondary metabolites through ribosomal and non-ribosomal pathways, including lipopeptides (LPs) (surfactins, fengycins, iturins, and bacilysins), bacteriocins (acylatins and amyolysins), and polyketones (difficidins, bacillaenes, and macrolactins) [6, 9]. Bacillus lipopeptides are metabolite molecules produced by Bacillus that comprise a hydrophobic β-fatty acid chain and a hydrophilic ring structure with 7–10 amino acids [10]. The molecules exhibit excellent bio-active properties including broad-spectrum antibacterial activity, good stability, low toxicity, high biodegradability, and reduced drug resistance [11, 12]. Varying lengths of fatty acids lead to each lipopeptide family containing variants with different amino acid substitutions and isoforms [13]. Bacillus lipopeptides are primarily classified into three categories including surfactins, fengycins, and iturins [14]. Surfactin proteins comprise a heptapeptide moiety linked to β-hydroxylated fatty acids that form a cyclic lactone ring [15]. Surfactin proteins can disrupt the formation of biofilms and exert antiviral and antimycoplasma activities [16]. Iturins exert strong bactericidal activity and comprise a ß-amino fatty acid and seven α-amino acids [17]. Fengycin is a cyclic lipopeptide that contains a side chain of ß-hydroxy fatty acids with 16–19 carbon atoms and that is active against filamentous fungi [18]. Further, when different families are co-produced, they can exhibit synergistic interactions and enhanced activities [19]. Lipopeptides produced by Bacillus exhibit specific insecticidal activities that can affect various insect orders including *Hemiptera* [20], *Lepidoptera* [21, 22], *Diptera* [23, 24]. Thus, Bacillus may represent a promosing microbial resource with insecticidal potential.

*Acyrthosiphon pisum Harris* is an insect of the family Aphididae and the order Homoptera. *(A) pisum* is distributed throughout China and feeds on the stems and leaves of plants using piercing-sucking mouthparts. The insect spreads various plant viruses, leading to significant agricultural economic losses. The lipopeptides produced by *(B) subtilis* possess localized insecticidal properties by interacting with cuticle molecules and inducing dehydration of aphid cuticles [1]. For example, the surfactin and iturin lipopeptides in the fermentation supernatant of *B. subtilis* both exhibited insecticidal activity against *Schizaphis graminum* [25]. Further, the fermentation supernatant of *B. velezensis* ATR2 exerted insecticidal effects on aphids, with various secondary metabolites including surfactin, bacillomycin, and fengycin identified in the supernatant through separation and purification [26]. Nevertheless, few studies have evaluated the mechanism underlying lipopeptide effects on bean tube aphids. The purpose of this study is to identify the insecticidal mechanism of the bean aphid by *B. velezensis*, including the separation and purification of insecticidal active substances to further the development of insecticidal control of bean aphids.

In this study, whole-genome sequencing and bioinformatics analysis of the highly effective aphidicidal strain *B. velezensis* ZLP-101 was conducted and secondary metabolite synthesis gene clusters of the strain were predicted. The crude extract of *B.velezensis* ZLP-101 was obtained by ammonium sulphate precipitation method and the insecticidal mechanism of strain ZLP-101 was revealed by analysing the changes in enzyme, tissue and behavior of aphids. Target compounds were isolated, purified, and characterized, followed by identification of the insecticidal-active components among the secondary metabolites of *B. velezensis* ZLP-101. These results provide a theoretical basis for the potential agricultural application of *B. velezensis* ZLP-101 and its insecticidal secondary metabolites.

## Results

### Whole-genome sequencing

#### *B. Velezensis* ZLP-101 genomic characteristics

**Fig. 1:**
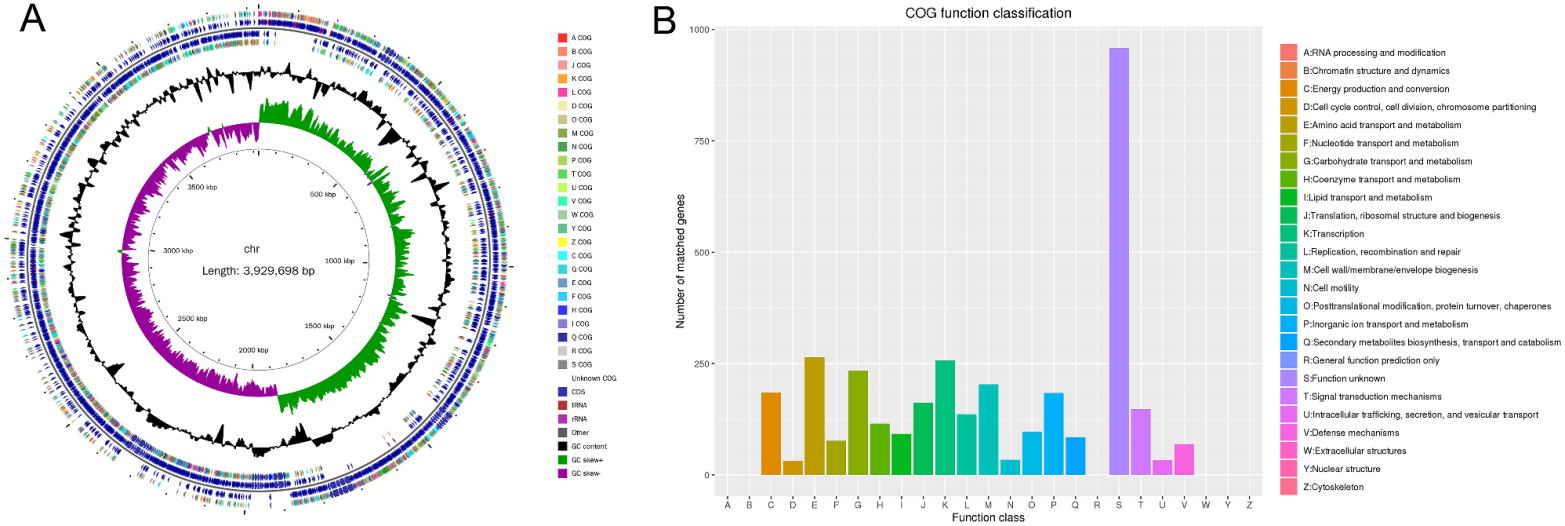
Genome circle map (**A**) and COG function annotation (**B**) of *B. velezensis* ZLP-101. (**A**) From inside to outside, circle (1) represents scale; circle (2) show GC skew; circle (3) represents GC content; circle (4) and circle (7) represent the COG to which each CDS belongs; circles (5) and circles (6) represent CDS, The location of tRNA and rRNA on the genome. (**B**) Functional classification of COG genes of strain ZLP-101

The whole genome of strain ZLP-101 was sequenced on the Illumina NovaSeq and Pacbio Sequel sequencing platforms, resulting in a total of 7,227,486 reads comprising 1,068,973,680 total base pairs, and approximately 271-fold coverage. The complete circular genome for strain ZLP-101 comprises 3,929,698 bp, with a GC content of 46.50% (Table 1; Fig. 1). The genome was predicted to encode 3,875 genes, 27 of which were rRNA operon genes, 82 were ncRNA operon genes, and 86 were tRNA genes. *De novo* predictions of repetitive sequences in the genome revealed 98 long terminal repeats, 27 long scattered repeats, 7 short scattered repeats, 44 transposons, and 4 satellite RNAs. Plasmid sequences were not identified in the genome assembly. Approximately 3,365 protein-coding genes of the genome were assigned COG number annotations within the five functional classes of the COG database that were primarily related to amino acid transport and metabolism, transcription, and carbohydrate transport metabolism.

**Table 1:** Genomic characteristics of *B. velezensis* ZLP-101

Attributes	Value
Genome size (bp)	3,929,698
G + C content (%)	46.50%
Plasmid	0
Predicted genes	3875
ORF num	3889
rRNA genes	27
tRNA genes	86
ncRNAs genes	82
CRISPRs	0

#### KEGG functional annotations of genes

**Fig. 2:**
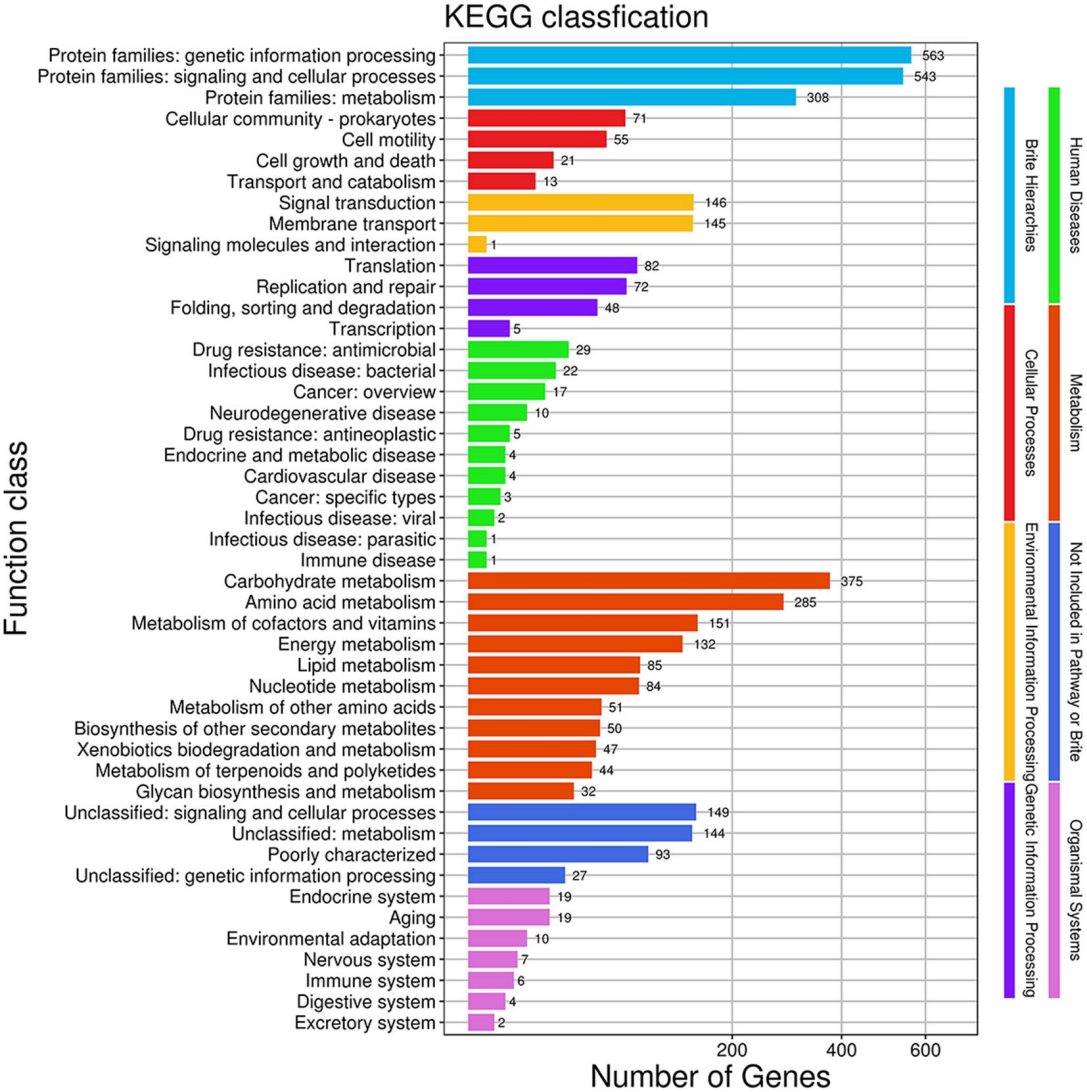
Functional annotation of secondary metabolite-related genes in *B. velezensis* ZLP-101 using KEGG metabolic pathway analysis

The *B. velezensis* ZLP-101 genome was annotated by comparison to the KEGG database, and the abundances of secondary functional classifications were summarized. Within the strain ZLP-101 genome, metabolism-related genes (1,336) were most prevalent, followed by environmental information processing-related (292), genetic information processing-related (207), and cell process-related (160) genes (Fig. 2). Diverse metabolism and energy metabolism pathways were identified that could support the nutrition and energy requirements for strain growth. Among them, 85 genes were involved in lipid metabolism, 50 in the biosynthesis of other secondary metabolites, and 44 in the metabolism of terpenoids and polyketides. Among the genes related to environmental information processing, 145 and 146 were involved in membrane transport and signal transduction, respectively. The annotation analysis suggested that the strain may have the potential to synthesize diverse lipopeptides, including potentially novel secondary metabolites. Many membrane transport-related genes are encoded by strain ZLP-101, indicating a potentially strong ability to secrete products. Overall, the results indicated that strain ZLP-101 exhibited a potentially high capacity for the synthesis of secondary metabolites.

#### GO functional annotation of genes

**Fig. 3:**
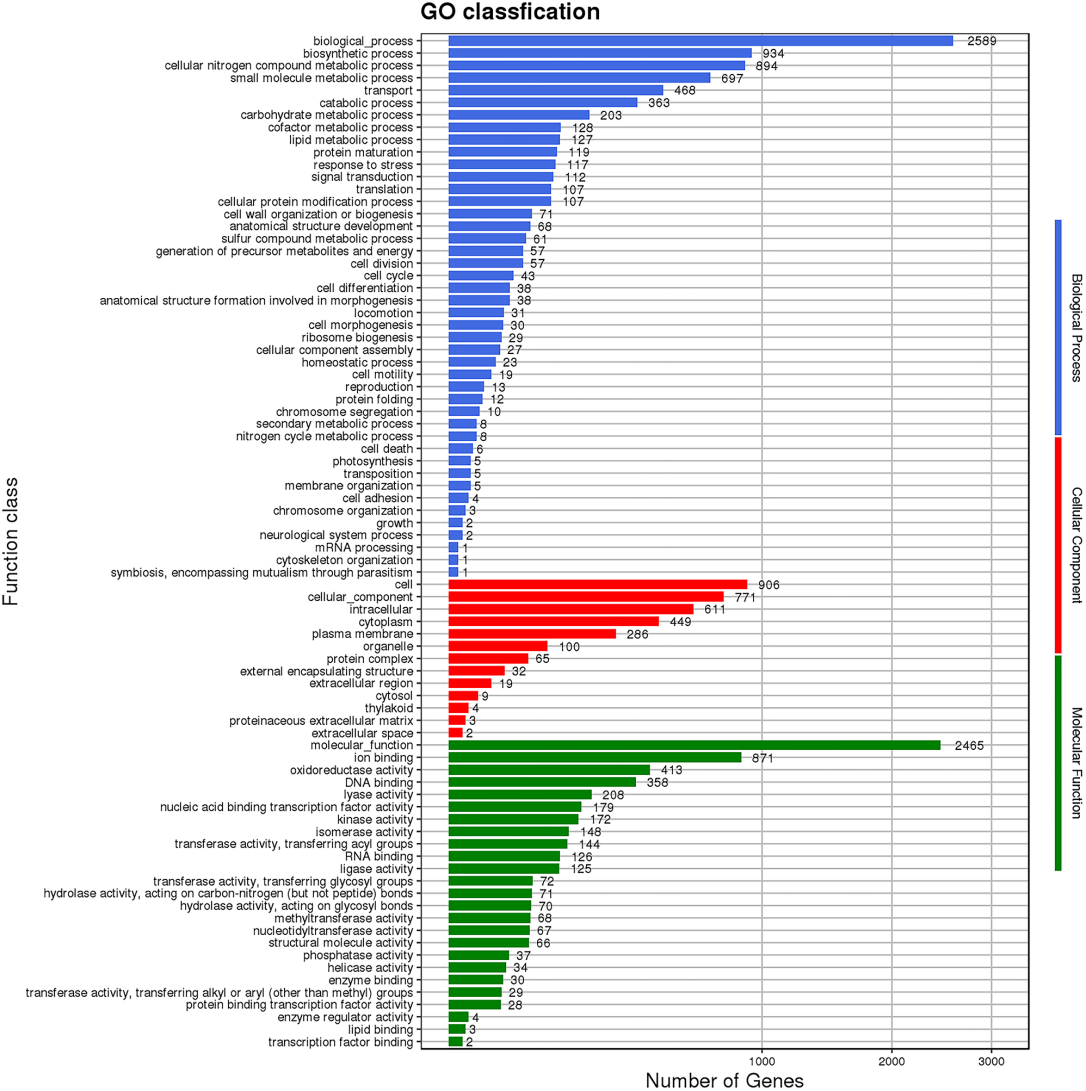
Go functional classification of *B. velezensis* ZLP-101 genes

GO functional analysis led to the annotation of 7,643, 3,257 and 5,790 genes within the GO categories of biological process, cellular components, and molecular functions, respectively (Fig. 3). The biological process and biosynthetic process categories comprise important functions in strain ZLP-101 physiology. Within the category of molecular function, ion binding, molecular function, and oxidoreductase activity were particularly enriched. Within the cellular components broader category, cellular parts and cellular components were the main functional sub-categories. Genes annotated to these categories may be involved in the synthesis and transport of secondary metabolites. Further, the abundances of genes annotated to these categories were relatively high, suggesting that strain ZLP-101 exhibits a molecular capacity to synthesize various secondary metabolites.

#### CAZy functional annotation of genes

**Fig. 4:**
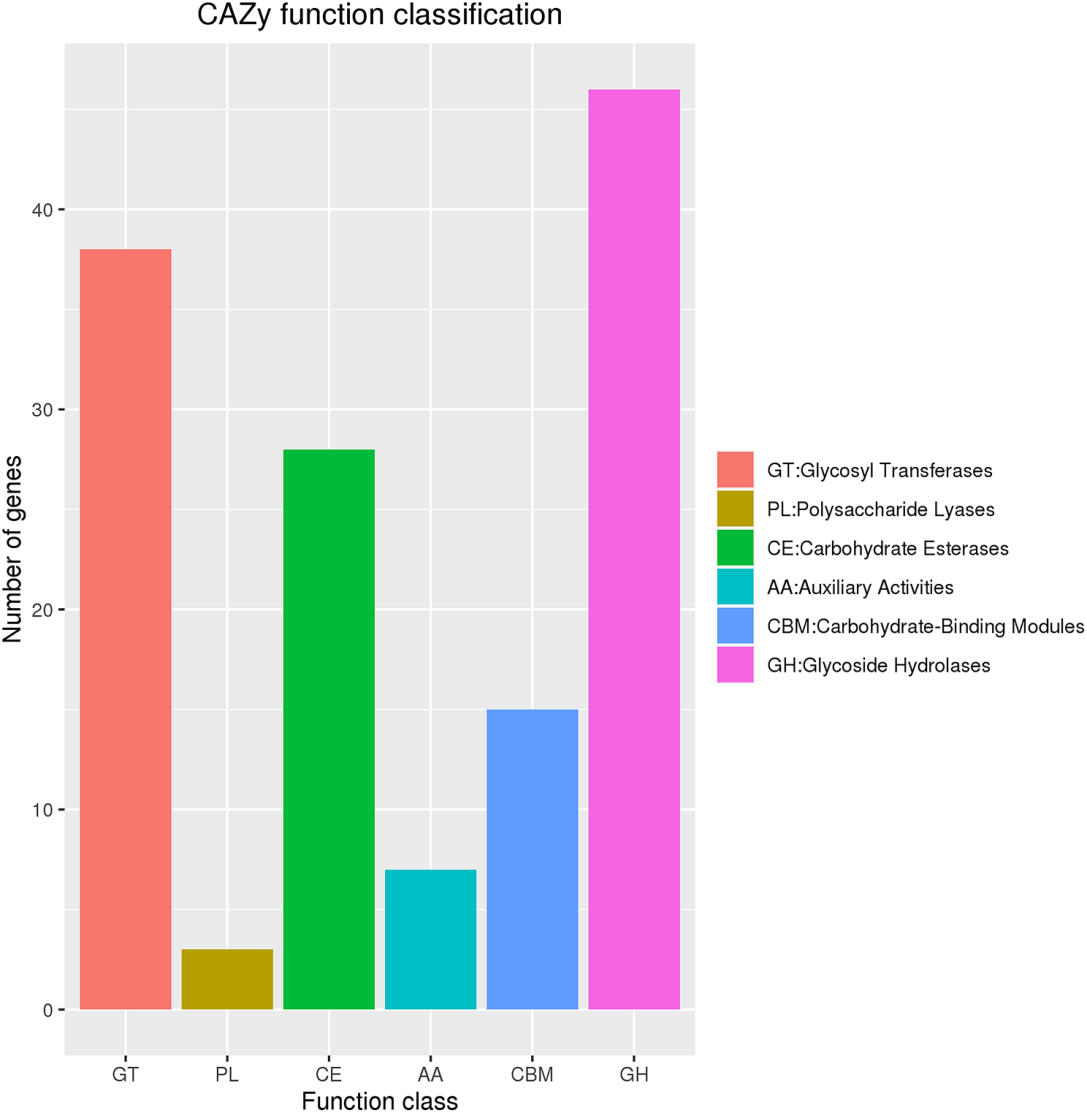
Gene distribution of CAZy family in the *B. velezensis* ZLP-101 genome

The CAZy enzyme database comprises functions related to carbohydrate degradation, synthesis, and modification. Consequently, genes from strain ZLP-101 were annotated against the database (Fig. 4). A total of 46 genes were identified that encoded glycoside hydrolases (GHs) in the strain ZLP-101 genome, in addition to 38 glycosyltransferases (GT), 28 carbohydrate esterases (CEs), and 15 carbohydrate binding modules (CBMs). Seven genes encoded accessory activities (AAs), while at least 3 genes encoded polysaccharide lyases (PLs). Thus, proteins related to glycoside hydrolases, glycosyltransferases, and glycolipases comprised a large proportion of carbohydrate-active enzymes, suggesting the presence of abundant structurally rich glycosyl-skeleton compounds as metabolites of strain ZLP-101. In addition, the detailed CAZy annotations for the strain ZLP-101 genome provide a research framework to further identify novel carbohydrate-active enzymes.

#### Prediction and identification of secondary metabolite gene clusters

Twelve secondary metabolite gene clusters were predicted in the genome of *B. velezensis* ZLP-101 using antiSMASH (version 7.0; Table 2). Differences in gene clusters encoding secondary metabolite synthases can be used to divide them into categories of NRPS, PKS-like, terpene, lanthipeptide-class-ii, transAT-PKS, and other types. The 6 gene clusters were identical to those known for macrolactin, bacillaenes, fengycins, difficidins, bacillibactins, and bacilysins, all of which exhibited 100% nucleotide similarity. Gene clusters 1 and 2 were similar to surfactin (82% similar) and butirosin (7% similar) gene clusters. In addition, 4 novel clusters were predicted, wherein clusters 3, 4, 8, and 9 did not exhibit any matches to known clusters.

**Table 2:** Secondary metabolite clusters encoded by the *B. velezensis* ZLP-101 genome

Cluste	Type of secondary metabolite	Length (bp)	Most similar known biosynthetic gene cluster (percent of similarity)	MIBiG BGC-ID
Cluster 1	NRPS	63,978	Surfactin(82%)	BGC0000433
Cluster 2	PKS-like	41,245	Butirosin A/Butirosin B(7%)	BGC0000693
Cluster 3	Terpene	17,409	NA	NA
Cluster 4	Lanthipeptide-class-ii	288,889	NA	NA
Cluster 5	TransAT-PKS	90,835	Macrolactin H(100%)	BGC0000181
Cluster 6	TransAT-PKS, T3PKS, NRPS	100,565	Bacillaene(100%)	BGC0001089
Cluster 7	NRPS, transAT-PKS, betalactone	134,316	Fengycin(100%)	BGC0001095
Cluster 8	Terpene	21,883	NA	NA
Cluster 9	T3PKS	41,100	NA	NA
Cluster 10	TransAT-PKS	93,792	Difficidin(100%)	BGC0000176
Cluster 11	NRPS, RiPP-like	51,791	Bacillibactin(100%)	BGC0000309
Cluster 12	other	41,418	Bacilysin(100%)	BGC0001184

### Insecticidal activity of strain ZLP-101 crude extract

#### Determination of the crude extract insecticidal LC50

The prepared 10 g/L crude extract aqueous solution was diluted to 625, 500, 416.7, 357.1, and 312.5 mg/L for insecticidal activity analysis using the 2-fold dilution method (Table 3). At 625 mg/L crude extract exposure, the aphid mortality rate was 98.67%. Increased dilution led to decreased aphid mortality rates. At 416.7 mg/L crude extract exposure, aphid mortality was 55.33%. The dilution of the crude extract was further refined to measure insecticidal activity. The SPSS software program (version 23.0) was used to calculate the LC50 value for bean aphids. The toxicity was evaluated via the equation y = 8.998x − 23.524, revealing an LC50 of 411.535 mg/L (*p* = 0.995), and indicating that the calculations were reliable.

**Table 3:** The insecticidal activity of crude extract aqueous solutions at different concentrations

Concentration, mg/L	Mortality,%	Concentration, mg/L	Mortality,%
625.00	98.67 ± 0.67	454.50	65.33 ± 3.06
500.00	86.67 ± 2.40	434.80	57.33 ± 3.06
416.67	55.33 ± 3.53	416.67	53.33 ± 4.16
357.14	34.67 ± 1.76	400.00	45.33 ± 5.03
312.50	13.33 ± 1.76	384.62	39.33 ± 4.16
CK1	3.33 ± 0.67	CK2	2.67 ± 1.15

*Note* The left side is for large-scale concentration exploration, while the right side is for refining small-scale concentration exploration

#### Effects of strain ZLP-101 crude extracts on aphid enzyme activities

**Fig. 5:**
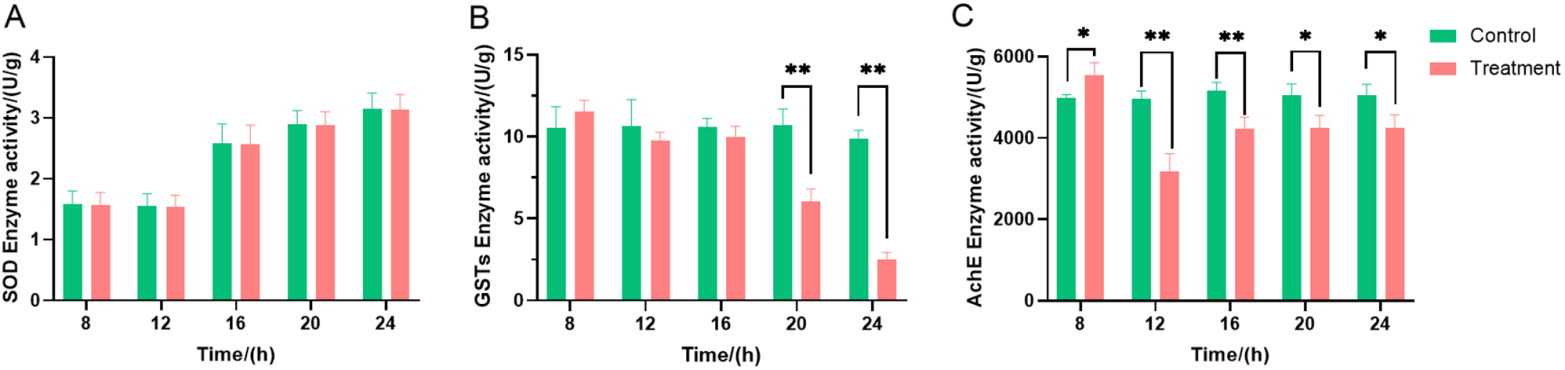
Effects of strain ZLP-101 crude extract on aphid enzymes. (**A**) Changes in SOD enzyme activity; (**B**) Changes in GSTs enzyme activity; (**C**) Changes in AchEs enzyme activity. The asterisks indicate a significant difference between aphids exposed to control and the corresponding crude extract aqueous solution (The experimental data were analyzed using one-way ANOVA, *: *p* < 0.05 and**: *p* < 0.01)

The effects of strain ZLP-101 crude extracts on aphid enzyme activities were evaluated (Fig. 5). Aphid SOD enzyme activities were inhibited to a certain degree, although not noticeably. Glutathione S-transferase plays an important role in physiological detoxification and metabolism. In the early stage of the experiment (i.e., the first 8 h) GST enzyme activity was higher in the treatment group than in the control group. With increasing time, GST enzyme activity became significantly lower in the treatment group than in the control group. In contrast, AchE activity decreased early in the treatment (i.e., the first 12 h) and then increased with increased treatment time.

#### Effects of strain ZLP-101 crude extract on aphid behaviors

**Fig. 6:**
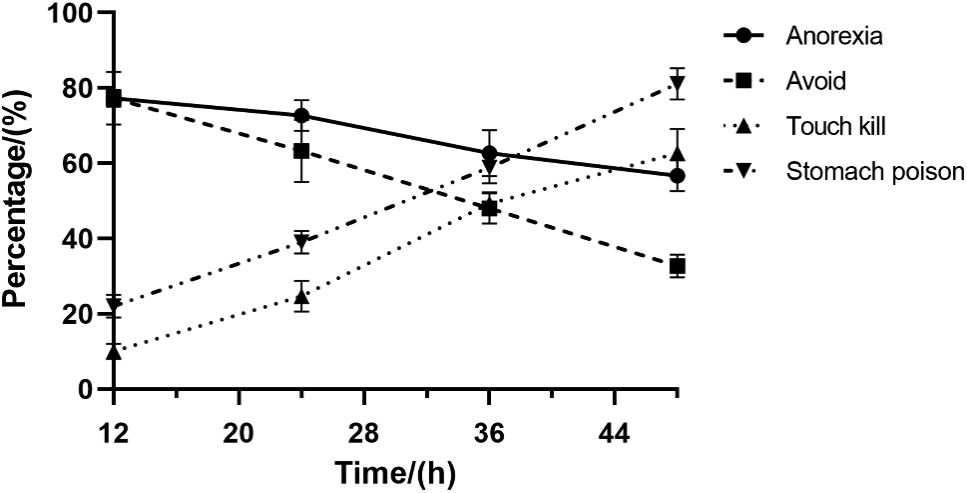
Aphid behavioral changes after exposure to strain ZLP-101 crude extracts

The crude extract of strain ZLP-101 exerted anti-feeding, avoidant, stomach poisoning, and contact killing effects on aphids (Fig. 6). The selective anti-feeding rates after 24 and 48 h of treatment were 71.51% and 57.29%, respectively, indicating strong anti-feeding effects due to insecticidal active substances, and that the anti-feeding rate decreased with time. The selective repelling rate of aphids after 12 h was 76.64%, indicating that the crude extract exerted a strong repelling effect on aphids during initial treatment. Increased time led to gradually weakened repellent activity of the crude extract against aphids. The mortality rate of aphids was 62.67% after 48 h of contact treatment with the crude extract, indicating an effect from contact to aphid body surfaces. The crude extract also affected aphids through stomach physiology, with the aphid mortality rate being 58.89% after 36 h of treatment. After 48 h of treatment, aphid death rates further increased, reaching 81.11%. In summary, the insecticidal active substances in strain ZLP-101 crude extract exert poisonous activities against aphids, but compared to contact killing of aphids, these effects are slow, with the mortality rate increasing with treatment time. Thus, stomach poisoning effects of the insecticidal active substances on aphids represent the primary factor related to insect death.

#### Effects of strain ZLP-101 crude extracts on internal aphid organs

**Fig. 7:**
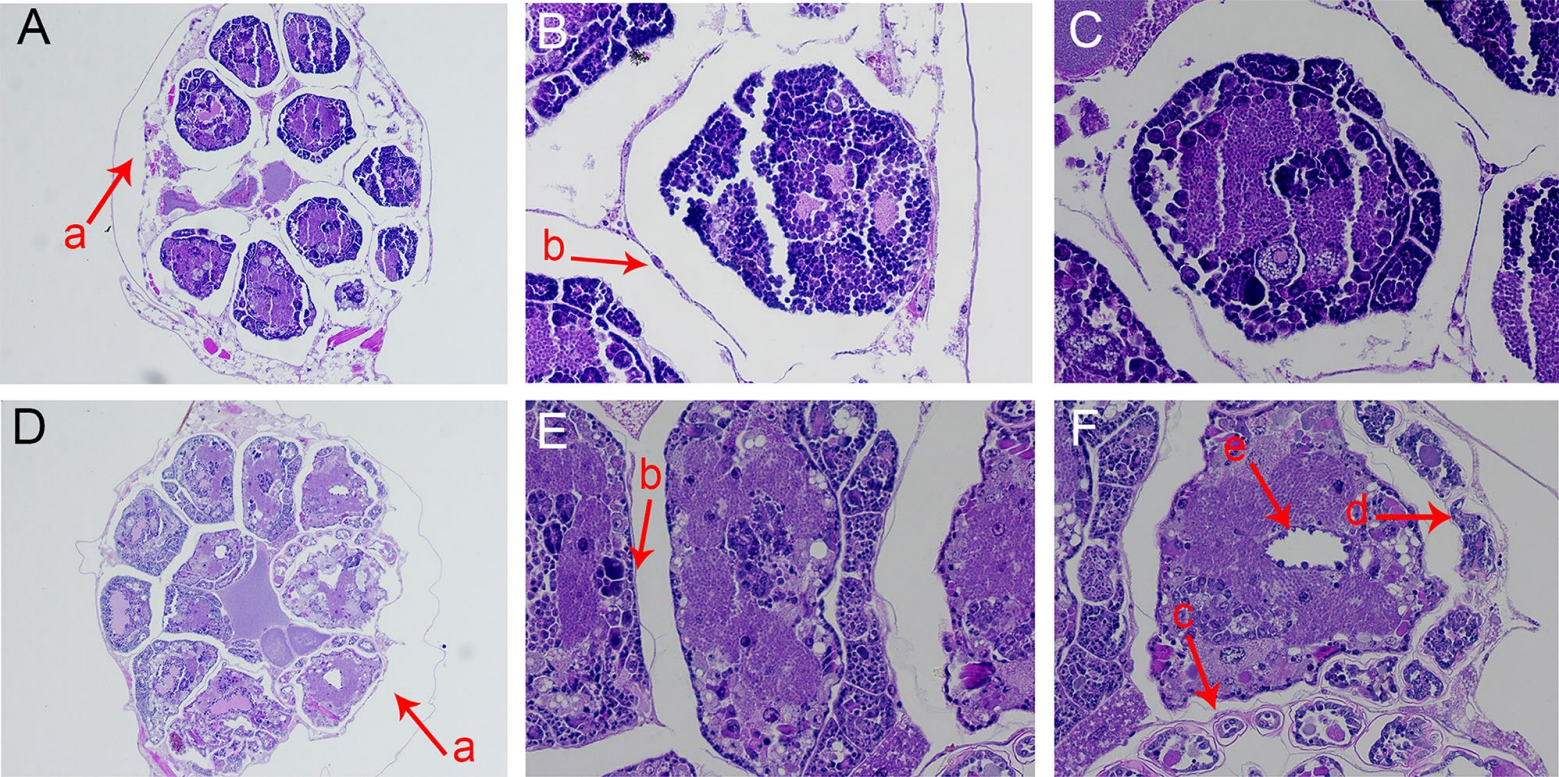
Tissue Sections of aphids treated with strain ZLP-101 crude extracts. Images A, B, and C are the control group sections. D, E, and F are the treatment group sections. A and D show 100x microscopy images. B, C, E, and F show 400x microscopy images. a, Expansion. b, Wall structure. c, Mesh. d, Deformation. e, Hole

The paraffin section method was used to cross-section aphid bodies, followed by light microscopy observations (Fig. 7). The cross-sections of bean aphids in the control group were regular, organs were neatly arranged, staining was uniform, and contents were encompassed by staining. The cross-sections of the treatment group aphids were irregular, contents were disorderly arranged with some parts overflowing, and the staining area was generally swollen (F-a), with no clear overall boundaries. Microscopic observations at 100x revealed that the edges of organs became thicker, without clear wall structures between organs (B-b). Observations at 400x revealed internal pores (F-e) in the organs, with a network of outer edges (F-c) and some severe distortions (F-d), indicating gradual worsening of lesions.

### Isolation and purification of insecticidal active substances from strain ZLP-101

**Fig. 8:**
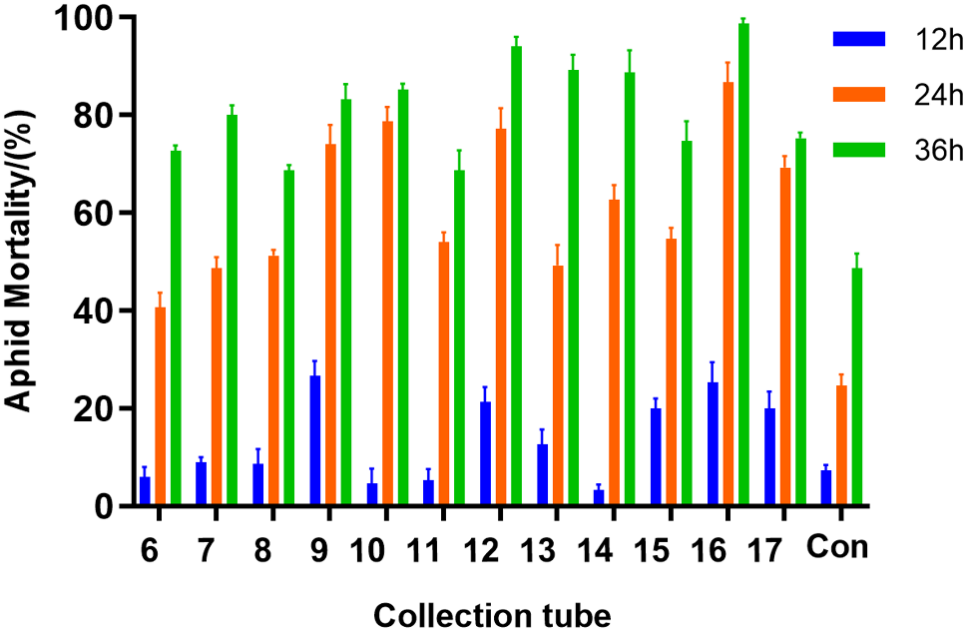
Insecticidal activity of semi-preparative components identified from crude extracts

In this study, the aphid lethality of 7 fractions reached more than 80% at 36 h (Fig. 8). The identification of these 7 fractions (7, 9, 10, 12, 13, 14 and 16) by LC-MS/MS revealed 15 aphidicidal active ingredients (Table S1).

Three iturin compounds were detected in this study in the range of m/z 1,044–1,094. The fragment ion peaks at [M + H]^+^(1,071.5852) [M + Na]^+^(1,093.5664) indicated the presence of C_16_iturin A/C_16_Mycosubtilin/ C_15_Bacillomycin F [27, 28]. Fragment ion peaks at [M + H]^+^(1,058.6698) [M + Na]^+^(1,080.6497) indicated the presence of C_15_iturin B. Peaks at [M + H]^+^(1,044.6546) [M + Na]^+^(1,066.6349) revealed C_14_iturin B (Figure S1) [3]. Mass spectrum identification of peaks at [M + H]^+^(1,022.67), [M + K]^+^(1,044.65) identified C_14_ surfactin A, while peaks at [M + H]^+^(1,036.69), [M + K]^+^(1,058.67), [M + Na]^+^(1,058.67) indicated C_15_ surface active factor A (Figure S2). These m/z values may also correspond to many other surfacting analogs that contain amino acid modifications at various positions in the peptide chains [29, 30]. Spergualin was also analyzed by secondary mass spectrometry, revealing molecular ion peaks at [M + H]^+^ (808.4138), [M + Na]^+^ (426.2069), and [M + K]^+^ (442.2069), enabling further identification of spergualin (Figure S3).

Nine fengycin compounds were detected in the range of m/z 1,449–1,501. The structures of fengycins A and B are different, with the sixth amino acid of the cyclic peptides being Ala (89.1 Da) and Val (117.1 Da), respectively [31]. Consequently, different fragment ion peaks are observed during mass spectrometry. Fragment ion peaks at m/z (966/1,080) and m/z (994/1,108) are usually characteristic fragment ions to identify fengycins A and B [31]. Four fragment ion peaks [M + H]^+^ (1,435.7740, 1,464.8063, 1,477.8207, and 1,491.8349) were detected using mass spectrometry. All 4 peaks exhibit characteristic fragment ion peaks of m/z (966/1080), indicating that they are C_14_ fengycin A, C_16_ fengycin A, C_17_ fengycin A, and C_18_ fengycin A, respectively. Similarly, mass spectrometry detected fragment ion peaks of [M + H]^+^ (1,463.8039, 1,491.8367, 1,505.8518, 1,475.8377). These 4 peaks exhibited characteristic fragment ion peaks at m/z (994/1108), indicating that they represented C_14_ fengycin B, C_16_ fengycin B, C_17_ fengycin B, and C_15_ fengycin B. In addition, fragment ion peaks at m/z(952/1,066) and m/z (980/1,094) were detected by secondary mass spectrometry as characteristic fragment ions for fengycin subtypes A_2_ and B_2_ [31]. The characteristic fragment ion peak m/z (980/1,094) was detected at fragment ion peak [M + H]^+^ (1,449.7872), indicating the presence of C_14_ fengycin B_2_ (Figure S4).

## Discussion

Microorganisms play important roles in agricultural biological control. Specifically, the development of microbial-based biocontrol agents to manage pests is a promising strategy to achieve sustainable agriculture. *Bacillus velezensis* is widely distributed in natural environments, exhibits a good capacity to resist environmental stress and colonize rhizosphere niches, and can secrete diverse secondary metabolites. These metabolites can directly inhibit pests, but also enhance the innate immunity of plants, leading to the widespread use of *B. velezensis* in the development of biopesticides in laboratory research or commercially [5, 32]. Partial or complete gene sequencing data for 606 *B. velezensis* genomes were available in the NCBI database as of May 2023. Liang et al. previously analyzed the genome of *B. velezensis* ATR2, observing diverse secondary metabolite synthesis gene clusters [26]. In this study, 8 gene clusters were identified in the strain ZLP-101 genome that were similar to those that produce known active substances, in addition to 4 potentially novel secondary metabolite gene clusters. Thus, *B. velezensis* ZLP-101 secondary metabolites may comprise diverse biologically active compounds.

Our previous experimental studies revealed that the fermentation broth of strain ZLP-101 exerted strong killing effects on aphids. In this study, the crude extract from strain ZLP-101 was prepared by ammonium sulfate precipitation to investigate its insecticidal mechanisms against aphids. Most studies have focused on anti-feeding, repellent, stomach toxicity, and contact toxicity properties of botanical insecticides. Recently identified nicotine insecticides have exhibited strong anti-feeding activities against aphids [33]. Consistent with these previous studies, the crude extracts evaluated in this study exerted strong anti-feeding and repellent effects on aphids. We hypothesized that the insecticidal active substances in the crude extracts continuously stimulate the anorexia neurons of the aphid mandibular suppository, thereby inhibiting signals for aphid feeding neurons and weakening aphid feeding behaviors. The repellent activity of insecticidal active substances against aphids gradually decreased with time, potentially because the beanstalks not impregnated with crude extract could not meet the survival requirements of aphids. Thus, some wingless adult aphids choose to use crude extract treated beanstalk segment of crude extract to maintain their own activities, while others continue to refuse to eat, and finally die of hunger. After 36 h of treatment, aphids were poisoned, with the percentage of dead insects peaking at 58.89%. After 48 h of treatment, poisoning increased, and the mortality rate reached 81.11%. The active insecticidal substances exerted toxicity against aphids, but the effects were relatively slow. Indeed, compared to contact-based mortality, stomach toxicity due to insecticidal active substances was the primary mode of death in aphids.

Aphids develop physiological adaptations to stressors and one of the most rapid response mechanisms to abiotic stress is via enzymatic systems through a rapid cascade of interacting antioxidants, in addition to oxidative and detoxifying enzyme [34]. Under normal physiological conditions in insects, these enzymes and free radicals remain balanced, while under stressful conditions such as exposure to pesticides, pathogens, microsporidia, and extreme temperatures, the enzyme and free radical levels may change, disrupting this balance [35]. Stimulation by the synthetic plant hormone 1,1-dimethylpiperidine chloride (DPC) leads to rapid increases in the activities of SOD, POD, CAT, and GST in *Aphis gossypii*, while CarE and AchE activities decreased [36]. SOD plays important roles in balancing intracellular environments when insects face stresses [37]. Previous analysis of the crude extract from *Chelidonium majus* treatment of *Lymantria dispar* larvae revealed significantly inhibited SOD activity after 48 h, but significant activation at 72 h [37]. In this study, SOD activity in aphids treated with the crude extract of strain ZLP-101 was not clearly different to that of the control at 24 h, and the experimental treatment could be observed even later. GST exerts a pivotal role in insecticide detoxification within insects. Specifically, GST can combine with insecticidal molecules via chelation, or remove lipid metabolites induced by insecticidal compounds, thereby protecting tissues from oxidative damage [38]. Investigation of the contact toxicity of Pomelo seed oil to cowpea aphids (*A. craccivora*), revealed that GST levels in the aphids first increased and then decreased [38]. In this study, GST activity decreased with increasing treatment time. The insecticidal active substances of the crude extracts were hypothesized to damage the interior of aphids, which could then not produce enough detoxification enzymes and leading to their eventual death. AchE within the central nervous system is the target of organophosphate and carbamate insecticides, because its role in cholinergic synapse physiology is essential for organisms [37]. Triterpenoid saponins isolated from *Clematis lasiandra* did not exhibit any effects on AchE activity of pea aphids (*Acyrthosiphon pisum*) [39]. In a different study, *Abrus precatorius* extract exposed to cabbage aphids for 24 h, led to significantly inhibited AchE activity compared to controls. Further, AChE activity gradually increased after 48 h of treatment, but was generally lower than in the control group [40]. In this study, aphid AchE enzyme activity first decreased, then increased after treatment with strain ZLP-101 crude extracts. In the early stage of treatment, the AchE enzyme activity of the treatment group aphids were significantly lower than control group, and the AchE enzyme activity was inhibited to a certain extent. Prolonged treatment led to the gradual increase of AchE enzyme activity to levels similar to those of the control group. Overall, the insecticidal active compounds of strain ZLP-101 affects the protective enzymes and detoxification enzymes of aphids, destroys internal tissues of the aphids, and inhibits them from maintaining normal physiological activities, leading to their death.

To further understand the insecticidal active compounds in the secondary metabolites of strain ZLP-101 that can kill bean aphids, ammonium sulfate precipitation and reversed-phase chromatography were used to obtain seven cell-free components with strong insecticidal activity from supernatants, comprising 15 total compounds with strong insecticidal activity. Comparison to published LC-MS/MS data revealed that the active compounds were surfactins, iturins, fengycins, and spergualin. The iturins and spergualin could not be paired with known compounds in the antiSMASH software program. Lipopeptides are a mixture of isomers that may differ in peptide ring composition, but also most commonly differ in the length of fatty acid chains [20]. The surfactin compounds identified in this study were C_15_Surfactin A/C_16_Surfactin B and C_14_Surfactin A/C_15_Surfactin B/C_14_ Surfactin C. Surfactin has been shown to induce the death of insects from different orders including Lepidoptera, Diptera and Homoptera, indicating that it is a particularly promising insecticidal candidate [1, 20, 41]. The Iturin family identified in this study were C_16_Iturin A/ C_16_Mycosubtilin/ C_15_Bacillomycin F, C_14_ Iturin B, and C_15_Iturin B. Other studies have shown that iturin exerts insecticidal effects on *Spodoptera litura* [42]. Spergualin is a lipopeptide antibiotic synthesized in a non-ribosomal pathway and that exhibits antitumor and antibacterial activities [43, 44]. Nine fengycins were also identified in this study, including C_14_, C_16_, C_17_, and C_18_ fengycin B, in addition to C_14_, C_16_, C_17_, and C_18_ fengycin A. C_15_ fengycin B contains a fatty acid double bond. Phenchanin exhibits certain insecticidal effects against *P. rapae*, by presumably penetrating the epidermal cells of larvae [22]. Further, the supernatants and fermentation broths of *Bacillus amyloliquefaciens* strains have been shown to exert activity against peach aphids, while homologs of the lipopeptides kurstakins, surfactin, iturines, and fengicines did not exhibit activity against the aphids, probably due to the presence of other bacterial metabolites [45]. Further, the lipopeptides plipastatin, mycosubtilin, and surfactin isolated from *Bacillus subtilis* have been shown to kill aphids alone or in combination, although the effects from surfactin alone were greater than those arising from combined effects [1].

In contrast to previous studies, sample 16 from this study contained surfactin, iturin, and fengycin. Aphid mortality after exposure to sample 16 was significantly higher than for samples 9, 10, 12, 13, and 14, which only contained fengycin elements, indicating a synergistic effect among the three compound types improved insecticidal effects. Insecticidal sample 9 contained C_14_ fengycin A, while sample 10 contained C_14_ derivatives of fengycin B. The mortality rate of aphids from sample 9 was lower than that of sample 10, indicating that the insecticidal activity of C_14_ fengycin A was higher than that of C_14_ fengycin B, which may be related to amino acid differences in the cyclic peptides, although further studies are needed to confirm this hypothesis.

## Conclusions

In conclusion, the genome of *B. velezensis* ZLP-101 was sequenced and annotated; based on the genome data, the aphicide active substances of this strain were isolated and identified. Fifteen insecticidal active compounds were identified by HPLC and MS, including iturins, fengycins, surfactins, and spergualins. The crude extract of strain ZLP-101 lipopeptide was able to influence the life activity of aphids through enzymatic, behavioural and organ morphological changes in the aphid body. Our results indicate that *B. velezensis* ZLP-101 can be used as an effective biocontrol agent in agriculture. Future research will be necessary to further investigate the insecticidal mechanism of this strain against aphids.

## Materials and methods

### Aphids, strains, and culture conditions

The *Acythosiphon pisum Harris* (hereafter, “aphids”) was used as the research object, and it was raised in the microbial aphid culture room of the Institute of Biology, Hebei Academy of Sciences. Rearing and experiments were performed in a growth chamber at 24 ± 2 °C, 60 ± 5% RH, with a 14:10 L: D cycle.

*B. velezensis* ZLP-101 is an efficient insecticidal strain selected from the biocontrol bacteria stored in our laboratory. Nutrient Brothtrain (NB) broth medium (containing 10 g/L peptone, 5 g/L beef extract, 10 g/L glucose, 5 g/L sodium chloride in distilled water, adjusted pH to 7.0) was used as growth medium. Bacteria were cultured at 32 °C for 16 h with continuous shaking at 180 r/min.

### Whole-genome sequencing

#### DNA extraction, genome sequencing, and assembly

Genomic DNA of *Bacillus velezensis* ZLP-101 was extracted using a bacterial genome kit. A library with a 10-kb insert size was constructed for sequencing. DNA sequencing was performed by Shanghai Pacbio Biotechnology Co., Ltd., and the whole genome of *B. velezensis* ZLP-101 was sequenced using an Illumina NovaSeq system and the third-generation high-throughput Pacbio Sequel sequencing technology. The quality statistics software AdapterRemoval (version 2.2.2) and SOAPec (version 2.03) were used to optimize the removal of connectors and low-quality sequences of second-generation sequencing raw data. The preliminary assembly results were obtained based on the three-generation sequencing data using the software HGAP (version v4) and CANU (version 1.7.1). The quality-filtered second-generation sequencing data were compared to the preliminary assembly results, and the assembly results were further corrected using Pilon v1.18 software to obtain the final assembly results. The complete genome sequence of *B. velezensis* ZLP-101 has been submitted to GenBank under the accession number CP128992.

#### Gene prediction and annotation

Protein sequences were predicted from the genome and annotations were identified using BLAST searches with the e-value threshold of the sequence alignments set to 1×e^− 5^. The best matches from searches were identified as the annotation for the gene. The sequences were annotated and matched to the databases for functional information, such as NCBI non-redundant protein (NR), Gene Ontology (GO), Kyoto Encyclopedia of Genes and Genomes (KEGG), Cluster of Orthologous Groups of Proteins (COG), and Carbohydrate-Active Enzymes (CAZy). Prediction and analysis of gene clusters encoding secondary metabolites were conducted using the antiSMASH software program (version 7.0).

### Preliminary identification of the insecticidal mechanism of strain ZLP-101

#### Preparation of ZLP-101 crude extracts

A 50 L fermenter was used to prepare fermentation broth from strain ZLP-101 that was then filter sterilized using a 0.22 μm filter membrane. Ammonium sulfate was added to the filtrate at a saturation concentration of 60%, and let stand at 4 °C for 7–8 h, followed by centrifugation at 8,000 r/min for 20 min. Supernatants were then discarded. The ammonium sulfate precipitate was placed in a pre-treated 1 kDa dialysis bag and placed in ultrapure water, followed by dialyzation at 4 °C for 48 h, with water exchanged every 6 h. The crude extract was then obtained after concentration by nitrogen blowing and freeze-drying.

#### Determination of the insecticidal LC50 of ZLP-101 crude extracts

ZLP-101 crude extract was prepared as an aqueous solution at a concentration of 0.01 g/mL that was diluted 16-, 20-, 24-, 28-, and 32-fold. Ultrapure water was used as a control. Fifty aphids of similar age and size were selected with a brush and placed in a Petri dish after they were subjected to starvation for 4 h. Broad bean stems of equal weight were soaked in crude extract aqueous solutions for 20 min, water was removed with filter paper, the stems were removed, and then placed on a pad in a Petri dish with filter paper. A pipette was then used to add 2 mL of deionized water to maintain moistness, followed by sealing with plastic wrap, and then piercing densely packed holes with a syringe needle. The bean stems were replaced every 12 h and the numbers of dead aphis were measured for different dilutions to calculate the mortality rate. Specifically, the aphid abdomens were gently touched with a brush and those that did not move were considered dead.

#### Effects of strain ZLP-101 crude extracts on aphid enzyme activities

To evaluate the enzyme activities of aphids, 10 mL of crude extract aqueous solutions at a concentration of 0.04 g/mL were prepared, with water used as a control, similar to the framework described in Sect. 2.3.2. Wingless adult aphids treated for 8, 12, 16, 20, and 24 h were stored at − 80 °C, with each treatment repeated in triplicate, and with about 100 aphids used per replicate. Treated aphids were rinsed three times with 0.9% NaCl aqueous solution and dried with filter paper, followed by weighing 0.1 g of aphids, adding 1 mL of extract and homogenizing in an ice bath. The samples were then centrifuged at 8,000 g for 10 min at 4 °C and the supernatants were removed and stored at − 80 °C. An enzyme activity assay kit was used to determine the activities of Superoxide dismutase (SOD), acetylcholinesterase (AchE), and glutathione-S-transferases (CSTs) (Beijing Suolaibao Technology Co., Ltd.), following the manufacturer’s instructions. Activity measurements were conducted in triplicate.

#### Strain ZLP-101 crude extract effects on aphid behavior

##### Anti-feeding behavior assays

To evaluate aphid behavior after exposure to strain ZLP-101 crude extracts, 10 mL of the extract aqueous solutions at a concentration of 0.02 g/mL were prepared, and bean stems cut to the same weight and roughly the same length were soaked in crude extract aqueous solution for 2 min. The excess solution was removed with filter paper and placed in culture medium covered with wet filter paper. Then, two stem segments were placed in each dish equidistant from the edge of the filter paper. Fifty aphids that were starved for 4 h were placed in the Petri dishes with the stems, with 150 insects evaluated overall. Plastic wrap was then used to seal the dishes and small holes were pierced in it with a needle. Then, 1 mL of ultrapure water was added every 12 h to maintain culture dish moistness. Water-treated broad bean stems were used as controls. Aphid abdomens were gently touched with a brush and those that did not move were considered dead. Food intake was measured every 12 h using the following formula:

1 $$\text{Selective}\,\text{refusal}\,\text{rate}=\text{(A-B)}/\text{(A+B)}\times100\%$$

Where A is the food intake of the control group; B is the food intake of the treatment group.

##### Determination of repellent effect

To determine the repellent effects of fermentation crude extracts, the same experimental methods were used as described in Sect. 5.3.3.1, except that four sections of bean stems of the same weight and approximately the same length were placed in each dish. Two sections were used for treatments and two for controls that were alternately placed in the plate, with the four stem sections equidistant from the edge of the filter paper. The number of aphids on the bean stems of treatment and control groups were recorded every 12 h. The formula is as follows:

2 $$\text{Selective}\,\text{avoidance}\,\text{rate}=\text{(A-B)}/\text{(B)}\times100\%$$

Where A is the average number of aphids in the control group; B is the average number of aphids in the treatment group.

##### Contact killing effects

To investigate killing effects, healthy wingless adult aphids of approximately the same size were placed in a Petri dish containing bean stems. Then, 5 µL of a 0.02 g/mL crude extract aqueous solution was pipetted onto the thoracic plate of the aphid. Observations were made every 12 h and the number of dead insects was recorded. Measurements were conducted with 3 replicates and 50 aphids per replicate, with water used for the control. The lethal effects were calculated as follows:

3 $$\text{Aphid}\,\text{Mortality}=\text{(Number}\,\text{of}\,\text{deaths}/\text{total)}\times100\%$$

##### Stomach toxicity

To investigate stomach toxicity, 10 mL of a crude extract aqueous solution was prepared at a concentration of 0.02 g/mL and used to soak bean stem segments for 2 min. The excess liquid was absorbed with filter paper and placed in a Petri dish covered with wet filter paper. Then, approximately 50 wingless adult aphids of the same size were placed in each dish that was then sealed with plastic wrap, followed by piercing the wrap with dense small holes using a syringe. Bean stems of the control group were soaked in water using the same volume. Water was added (1 mL) every 12 h and the stems were replaced, along with recording the number of dead aphids, using the methods described above. The death rate and the corrected death rate of aphids were then calculated.

#### Effect of ZLP-101 crude extracts on the internal tissue morphology of aphids

To evaluate tissue morphologies after exposure to crude extracts, wingless bean aphids with consistent growth and development were selected for experimentation. The treatment group was fed bean stems soaked in 0.04 g/mL crude extract aqueous solution for 2 min, while the control group was fed bean stems soaked in water for 2 min. The experiment was repeated in triplicate, with a total of 60 aphids. Live aphids from the treatment and control groups were taken after feeding for 24 h, then starved for 2–4 h, followed by treating 6 aphids from each group for paraffin sectioning, with 3 replicates total and 2 aphids in each replicate. The aphids were fixed with 4% paraformaldehyde/universal tissue fixative, stained with HE, and tissue sections were prepared. The paraffin sectioning and tissue staining methods were primarily conducted based on the method of Xiong Zhengyan described paraffin sectioning method for insect midguts [46].

### Isolation and purification of strain ZLP-101 secondary metabolites

The crude extract was prepared according to the same experimental methods were used as described in Sect. 5.3.1. Further purification was carried out by HPLC(SHIMADZU LC-20 A, Japan) with a C18 column (250 × 4.6 mm, 5 μm; WONDASIL, Japan) at room temperature. The mobile phase consisted of acetonitrile and HPLC-grade water (with 0.1% trifluoroacetic acid [TFA]). A linear gradient was used for elution at a flow rate of 1 ml/min as follows: 0–60 min, from 40 to 90% acetonitrile (linear gradient); 60–66 min, 90% acetonitrile (isocratic); 66–68 min, from 90 to 40% acetonitrile (linear gradient); 68–78 min, 40% acetonitrile (isocratic). Elution was monitored by determining absorbance at 214 nm, and fractions were manually collected each minute. Fractions with aphidicidal activity were further screened for structural identification.

### LC-MS/MS analysis

The samples with insecticidal activity were analyzed by mass spectrometry using an AB SCIEX X500R QTOF system (Beijing Omico Biotechnology Co., Ltd). The conditions of UPLC were as follows: mobile phase A was acetonitrile containing 0.1% (volume ratio) formic acid, and mobile phase B was HPLC-grade water containing 0.1% (volume ratio) formic acid, Waters UPLC HSS T3 (C18; 2.1 mm×100 mm, 1.7 μm), 30-90% acetonitrile linear gradient for elution for 20 min, 90% acetonitrile isovolumic for elution for 10 min, 90%-30% acetonitrile linear gradient for elution for 3 min, 30% acetonitrile isovolumic for elution for 10 min. The injection volume is 5.0 µL, the flow rate was 0.3 mL/min, the detection wavelength was 214 nm, and the column temperature was 40 °C. The mass scanning range was 50-4000 M/Z, the capillary voltage was 3000 V, and the desolvent temperature is 400 °C.

### Electronic supplementary material

Below is the link to the electronic supplementary material.

Supplementary Material 1

## Data Availability

All data and material are available upon request to the corresponding author.
