# Supplementary material for: Lipopeptides from Bacillus velezensis ZLP-101 and their mode of action against bean aphids Acyrthosiphon pisum Harris
Source: BMC Microbiol. 2024 Jun 29;24:231. doi: 10.1186/s12866-024-03378-2 (PMC11218388; doi:10.1186/s12866-024-03378-2)
Supplement: Supplementary file 1 — Supplementary Material 1 [file 12866_2024_3378_MOESM1_ESM.docx]

**Lipopeptides from *Bacillus velezensis* ZLP-101 and their mode of action against bean aphids *Acyrthosiphon pisum* Harris**

Qiuyue Liu^abc1^, Wenya Zhao^ac1^, Wenya Li^ac1^, Feiyan Zhang^ac^, Yana Wang^ac^, Jiangping Wang^abc^, Yumeng Gao^abc^, Hongwei Liu^ac*^, and Liping Zhang^ac*^

^a^Institute of Biology, Hebei Academy of Science, Shijiazhuang 050081, P.R. China

^b^Hebei Normal University, Shijiazhuang 050024, P.R. China

^c^Main Crops Disease of Microbial Control Engineering Technology Research Center in Hebei Province, Shijiazhuang 050081, P.R. China

*Corresponding author at: Institute of Biology, Hebei Academy of Science, Shijiazhuang 050081, P.R. China. E-mail addresses: lhwei1987@126.com (HW. Liu), lizzle-69@163.com (LP. Zhang).

^1^ These authors have contributed equally to this work.

**Supplementary material**

**Supplementary Figures**

**Supplementary Fig. S1**

**Figure S1. The mass spectrum of the iturin and its family analogs.**

(a) the primary mass spectrum (b) the secondary mass spectrum.

**Supplementary Fig. S2**

**Figure S2. The mass spectrum of surfactant active substance**.

(a)the primary mass spectrum (b)the secondary mass spectrum.

**Supplementary Fig. S3**

**Figure S3. The mass spectrum of the Spergualin active substance.**

(a) Spergualin primary mass spectrum (b) Spergualin secondary mass spectrum.

**Supplementary Fig. S4**

_2_

_2_

**Figure S4. The mass spectrum of fengycin and its derivatives.**

(a) the primary mass spectrum (b) the secondary mass spectrum.

**Table S1. Detection of m/z value of insecticidal compounds by LC-MS.**

| Fraction no. | Compound no. | Retention time(min) | Observed mass peak | | | Characteristic  fragment ions | | Identification |
| --- | --- | --- | --- | --- | --- | --- | --- | --- |
|  |  |  | [M+2H]^2+^ | [M+H]^+^ | [M+Na]^+^ |  |  |  |
| 7 | 1 | 7.876 | 536.2962 | 1,071.5852 | 1093.5664 | - | - | C_16_IturinA/C_16_Mycosubtilin/C_15_BacillomycinF |
|  | 2 | 9.209 | 404.2069 | 808.4138 | 426.2069 | - | - | Spergualin |
| 9 | 3 | 9.562 | 718.3902 | 1,435.7740 | - | 966.4569 | 1,080.5372 | C_14_Fengycin A |
| 10 | 4 | 10.257 | 732.4060 | 1,463.8039 | 1,485.7890 | 994.4905 | 1,108.5644 | C_14_Fengycin B |
|  | 5 | 10.290 | 725.3981 | 1,449.7872 | 1,470.8122 | 980.4715 | 1,094.5464 | C_14_Fengycin B_2_ |
| 12 | 6 | 11.446 | 732.4047 | 1,464.8063 | - | 966.4505 | 1,080.5260 | C_16_Fengycin A |
| 13 | 7 | 11.845 | 739.4130 | 1,477.8207 | - | 966.4257 | 1,080.5291 | C_17_Fengycin A |
|  | 8 | 12.277 | 746.4211 | 1,491.8367 | - | 994.4831 | 1,108.5590 | C_16_Fengycin B |
| 14 | 9 | 12.982 | 753.4283 | 1,505.8518 | - | 994.4831 | 1,108.5586 | C_17_Fengycin B |
| 16 | 10 | 14.259 | 738.4222 | 1,475.8377 | - | 994.4843 | 1,108.5596 | C_15_Fengycin B |
|  | 11 | 13.969 | 746.4201 | 1,491.8349 | - | 966.4567 | 1,080.5313 | C_18_Fengycin A |
|  | 12 | 21.800 | - | 1,036.6878 | 1,058.6698 | - | - | C_15_Surfactin A/C_16_Surfactin B |
|  | 13 | 21.861 | - | 1,058.6698 | 1,080.6497 | - | - | C_15_Iturin B |
|  | 14 | 24.858 | - | 1,022.6725 | 1,044.6546 | - | - | C_14_Surfactin A/C_15_Surfactin B/C_14_Surfactin C |
|  | 15 | 24.980 | - | 1,044.6546 | 1,066.6349 | - | - | C_14_Iturin B |
